# KI and WU Polyomaviruses in Patients Infected with HIV-1, Italy

**DOI:** 10.3201/eid1508.090424

**Published:** 2009-08

**Authors:** Muhammed Babakir-Mina, Massimo Ciccozzi, Elisabetta Trento, Carlo Federico Perno, Marco Ciotti

**Affiliations:** Foundation University Hospital Tor Vergata, Rome, Italy (M. Babakir-Mina, C.F. Perno, M. Ciotti); Istituto Superiore di Sanità, Rome (M. Ciccozzi); Istituto di Ricovero e Cura a Carattere Scientifico Instituto Fisioterapico Ospetaliere–San Gallicano Institute, Rome (E. Trento)

**Keywords:** Viruses, polyomavirus, HIV-1, Italy, letter

**To the Editor:** Before 2007, two human polyomaviruses were known to infect humans: BK virus and JC virus (*1*,*2*). Recently, 2 novel polyomaviruses, KI polyomavirus (KIPyV) and WU polyomavirus (WUPyV), were identified in the respiratory secretions of children with signs of acute respiratory signs (*3*,*4*); little evidence exists to suggest that these viruses are causative agents of respiratory tract disease (*5*). To determine the prevalence of WUPyV and KIPyV in the plasma of HIV-1–infected patients, we screened 62 persons who were HIV-1 positive by using PCR to detect the 2 viruses. We also conducted phylogenetic analysis of the identified strains.

Plasma specimens were collected at Istituto di Ricovero e Cura a Carattere Scientifico Instituto Fisioterapico Ospetaliere–San Gallicano Institute and Tor Vergata University Hospital, Rome, Italy, from April 2005 through September 2008. Patients were adults (37–54 years of age, median age 45.5 years) and were being treated with antiretroviral drugs. HIV-1 viral load determination, CD4+ counts, and HIV-1 genotyping were performed as part of the routine investigation. Plasma viremia levels ranged from <50 to 2,877,764 copies/mL, and CD4+ counts ranged from 150 to 1,218. Most patients (64.5%) were infected by HIV-1 subtype B. Other subtypes found were F, G, and C.

Total DNA was extracted from 0.2 mL of plasma by using the QIAamp DNA Mini Kit according to the manufacturer’s instruction (QIAGEN S.p.A., Milan, Italy) and then stored at –80ºC until analysis. KIPyV and WUPyV PCR screening was carried out as described (*3*,*4*). Positive isolates were reamplified with primers encompassing the N-terminal part of the large T antigen (*T-Ag*) and almost the entire small t antigen (*t-Ag*) genes. KIPyV was amplified as described (*6*), and, for WUPyV, the primers were FWUV4460 5′-ACTGAGACCACCAGTAATCCCAG-3′ (4460–4482 nt) and RWUV5200 5′-AAGCAGAGGGCCTTGCTGAGGCG-3′ (5200–5178 nt). The thermal cycling profile was 1 cycle at 94ºC for 10 min and then 40 cycles at 94ºC for 30 s, at 65ºC for 30 s, and at 72ºC for 60 s. The amplified *t-Ag* fragments were sequenced as described (*6*). The obtained sequences (KIV-RM21, KIV-RM22, and WU-IT3) were submitted to GenBank (accession nos. FJ842112–FJ842114) and matched against all deposited sequences (www.ncbi.nlm.nih.gov/BLAST). ClustalX software (http://bips.u-strasbg.fr/fr/documentation/clustalx/#g) was used to obtain alignment with a set of KIPyV and WUPyV isolates from Italy (*6*,*7*) (accession nos. FJ389513–FJ389516, FJ594120–FJ594126, FJ594118, FJ594119, FJ804123, FJ811519–FJ811524, FJ824854, and FJ821706) and prototype strains for KIPyV (EF127906, EF127908, and EF520288) and for WUPyV (EF444549–EF444554, EU711054–EU711058, EU296475, EU358768, and EU358769). Alignment was manually edited with the Bioedit software (www.mbio.ncsu.edu/BioEdit/bioedit.html) (*6*). Positions containing gaps were removed from the final alignment. For our dataset, the best fitting nucleotide substitution model was tested with a hierarchical likelihood ratio test following the strategy described (*6*),which used a neighbor-joining (NJ) base tree with LogDet-corrected distances (http://paup.csit.fsu.edu/about.html). Maximum-likelihood (ML) trees were then inferred with the selected model and ML-estimated substitution parameters. The heuristic search for the ML tree was performed by using an NJ tree as starting tree and the tree–bisection-reconnection branch-swapping algorithm. NJ trees were also estimated by using pairwise distances inferred by ML with the best fitting nucleotide substitution model. Calculations were performed by using PAUP* 4.0b10 (http://paup.csit.fsu.edu/about.html) (*6*). Statistical support for internal branches in the NJ trees was obtained by bootstrapping (1,000 replicates) and with the ML-based, zero–branch-length test for the ML trees (*6*).

Of 62 plasma specimens screened, PCR detected KIPyV in 2 (3.2%) and WUPyV in 1 (1.6%). All 3 patients were infected by HIV-1 subtype B. Phylogenetic analysis of the *t-Ag* of the 3 isolates showed that KIV-RM21 and KIV-RM22 are not closely related to the KIPyVs isolated in Italy from feces (KIV-RM5 to KIV-RM11), respiratory tract (KIV-RM1 to KIV-RM4), and tonsils (KIV-RM12 to KIV-RM20), nor are they related to those previously identified (*6**,**7*). Similarly, WUV-IT3 was not related to WUV-IT1 or WUV-IT2 nor to WUPyVs identified in stool and respiratory tract secretions (Figure).

**Figure:**
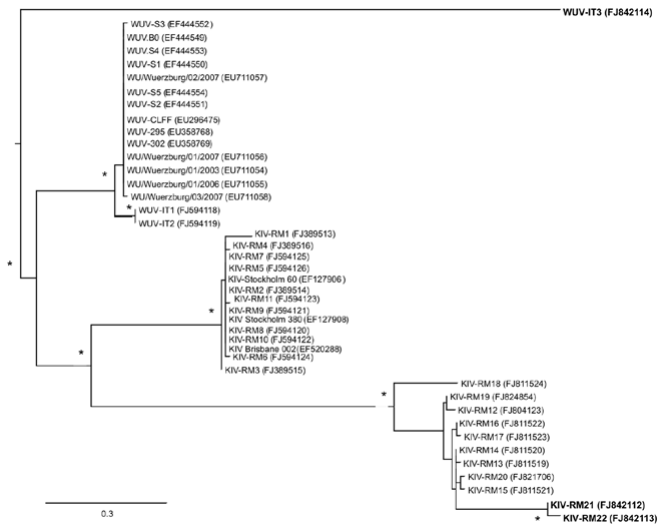
Unrooted phylogenetic tree showing analysis of KI (KIV-RM21, KIV-RM22) and WU (WUV-IT3) polyomaviruses (KIPyVs, WUPyVs, respectively) identified in the plasma of HIV-1–positive patients. The identified strains are indicated in **boldface**, and the phylogenetic analysis refers to the small t region. The other polyomaviruses shown in the figure are the KIPyVs (KIV-RM1 to KIV-RM20) and WUPyVs (WUV-IT1 and WUV-IT2) identified in Italy in previous studies (*6**,**7*) and the prototype strains for KIPyV (GenBank accession nos. EF127906, EF127908, EF520288) and WUPyV (GenBank accession nos. EF444549–EF444554, EU711054–EU711058, EU296475, EU358768, and EU358769). GenBank accession numbers for all virus strains are shown in parentheses. Multiple nucleotide sequence alignments were performed by using ClustalX software (http://bips.u-strasbg.fr/fr/documentation/clustalx/#g), and the phylogenetic tree was constructed by using the neighbor-joining algorithm with LogDet-corrected distances (http://paup.csit.fsu.edu/about.html) (*8*). An asterisk (*) beside a branch represents significant statistical support for the clade subtending that branch (p<0.001 in the zero-branch–length test) and bootstrap support >75%. Scale bar indicates nucleotide substitutions per site.

To date, KIPyV and WUPyV have been detected in respiratory secretions and stool and serum specimens from pediatric patients with acute respiratory symptoms and have been found in respiratory tissue of adults and children (*3*,*4*,*6*,*8*). Few data are available on the detection and reactivation of these novel polyomaviruses in immunocompromised patients (*9*,*10*). In this study, KIPyV and WUPyV sequences were found in 3.2% and 1.6% of HIV-1–infected patients, respectively. None of the patients had respiratory symptoms, so the presence of the 2 viruses in plasma raises the question of whether they play a pathogenic role in immunocompromised patients.

Molecular analysis of the KIPyV and WUPyV identified in plasma showed that these polyomaviruses were not closely related to strains identified previously in other countries nor to the KIPyVs and WUPyVs identified in Italy in stool, respiratory tract tissue, and tonsils. Whether this difference reflects a tropism of some strains for a particular tissue or organ remains to be established. Further studies are needed to clarify the possible pathogenic role of KIPyV and WUPyV in immunocompromised patients.
